# Intrinsic strong light-matter coupling with self-hybridized bound states in the continuum in van der Waals metasurfaces

**DOI:** 10.1038/s41563-023-01580-7

**Published:** 2023-06-22

**Authors:** Thomas Weber, Lucca Kühner, Luca Sortino, Amine Ben Mhenni, Nathan P. Wilson, Julius Kühne, Jonathan J. Finley, Stefan A. Maier, Andreas Tittl

**Affiliations:** 1 Chair in Hybrid Nanosystems, Nanoinstitute Munich, Faculty of Physics, Ludwig-Maximilians-Universität München, Munich, Germany; 2 Walter Schottky Institut, Department of Physics, School of Natural Sciences, Technische Universität München, Garching, Germany; 3 School of Physics and Astronomy, Monash University, Clayton, Victoria Australia; 4 Department of Physics, Imperial College London, London, UK

**Keywords:** Metamaterials, Two-dimensional materials, Electronic properties and materials, Nanophotonics and plasmonics

## Abstract

Photonic bound states in the continuum (BICs) provide a standout platform for strong light-matter coupling with transition metal dichalcogenides (TMDCs) but have so far mostly been implemented as traditional all-dielectric metasurfaces with adjacent TMDC layers, incurring limitations related to strain, mode overlap and material integration. Here, we demonstrate intrinsic strong coupling in BIC-driven metasurfaces composed of nanostructured bulk tungsten disulfide (WS_2_) and exhibiting resonances with sharp, tailored linewidths and selective enhancement of light-matter interactions. Tuning of the BIC resonances across the exciton resonance in bulk WS_2_ is achieved by varying the metasurface unit cells, enabling strong coupling with an anticrossing pattern and a Rabi splitting of 116 meV. Crucially, the coupling strength itself can be controlled and is shown to be independent of material-intrinsic losses. Our self-hybridized metasurface platform can readily incorporate other TMDCs or excitonic materials to deliver fundamental insights and practical device concepts for polaritonic applications.

## Abstract (web summary)

The authors demonstrate strong coupling in bound state in the continuum metasurfaces on nanostructured bulk WS_2_ and exhibiting sharp resonances with tailored linewidths and controllable light-matter coupling strength.

## Main

Understanding and maximizing the interaction between light and matter in nanoscale materials is a central goal of nanophotonics. Resonant nanosystems have been investigated for the confinement and control of electromagnetic energy in sub-wavelength volumes, leading to breakthroughs in light harvesting^1^, optical waveguiding^2^ and emission control^3^. Coupling light to electronic excitations in solid-state materials is of particular interest because of the creation of hybridized photonic and electronic states, called polaritons, showing exciting properties such as Bose–Einstein condensation^4^ and superfluidity^5^ with potential for applications in low-threshold semiconductor lasers^6^, photocatalytic enhancement^7^ and quantum computing^8^. The driving force for research on excitonic coupling has been reaching the strong light-matter coupling regime, where coherent exchange of energy between photons and excitons takes place. Transition metal dichalcogenides (TMDCs) are a promising class of van der Waals materials for strong light-matter coupling, as they host strongly bounded excitons that are stable up to room temperature, owing to large binding energies >200 meV (ref. ^9^), coupled valley and spin degrees of freedom and broad tunability through strain, dielectric environment and the DC Stark effect^10–12^. TMDCs exhibit large excitonic oscillator strengths even in ~10–100 nm thick films^13^, rendering this class of materials compatible with established architectures of flat-optics devices to study strong light-matter interaction.

In general, reaching the strong coupling regime in nanophotonic systems requires high electromagnetic field intensities, confinement of light into small modal volumes and maximum overlap between the optical modes of the cavity and the excitonic resonances in the material. To date, strong coupling has been realized with TMDC monolayers in a variety of photonic systems, including plasmonic nanoantennas^14,15^, photonic crystals^16,17^ and distributed Bragg reflector microcavities^18^, as well as in self-hybridizing structures such as bulk TMDC slabs acting as Fabry–Perot cavity^19^ and single nanostructures supporting Mie resonances^20^.

Recently, photonic bound states in the continuum (BICs) have been shown to provide excellent control over light-matter interactions, owing to their strongly enhanced near fields, high quality factors and broad resonance tunability via variation of their geometrical parameters. Consequently, BICs have established themselves as essential new building blocks for nanophotonic vibrational spectroscopy^21,22^, light guiding^23^, harmonic generation^24^ and lasing^25^. Symmetry-protected metasurfaces^26^ have emerged as a prominent platform to realize BIC resonances for light-matter coupling applications, owing to their high optical signal contrasts even when subjected to fabrication imperfections^27^, and ease of optical measurements requiring only common brightfield microscopy setups. Importantly, the BIC metasurface concept provides direct control over the radiative decay rate (and therefore the linewidth) of the resonances via the asymmetry of the constituent unit cells^26^.

Currently, many implementations of excitonic strong coupling with BICs rely on placing TMDC layers adjacent to BIC metasurfaces composed of traditional all-dielectric materials such as Si, TiO_2_, Si_3_N_4_ or Ta_2_O_5_ (refs. ^17,28–31^). However, this approach increases system complexity due to the direct contact between different material systems and inhomogeneities introduced during the TMDC transfer process, where topographic irregularities and strain in the TMDC layers can lead to spectral shifts of the exciton^32^ or the suppression of non-linear effects^33^. Additionally, light-matter interaction occurs only via the evanescent fields around the BIC resonators, which decreases the coupling strength, especially when incorporating commonly used buffer layers for TMDCs, such as hexagonal boron nitride. Obtaining a TMDC BIC platform, which combines photonic cavity and excitonic material in the same nanostructured system, is therefore highly desirable; however, an experimental demonstration is still lacking.

Here, we realize precisely controlled strong coupling in self-hybridized WS_2_ metasurfaces based on symmetry-protected BICs. The metasurface platform consists of arrays of rod-type BIC unit cells, where the asymmetry of the structure is controlled via the length difference Δ*L* of the rods, offering direct control over the resonance linewidth. We experimentally demonstrate strong coupling with pronounced and tunable BIC resonances in WS_2_ metasurfaces fabricated via mechanical exfoliation, electron-beam lithography (EBL) and reactive-ion etching (RIE) (see Methods for more details). Resonance quality factors approach *Q* = 370 at spectral positions close to but spectrally separated from the exciton.

By varying the lateral size of the metasurface unit cells, the BIC resonances are tuned across the WS_2_ exciton, which reveals an anticrossing pattern with a Rabi splitting of 116 meV at ambient conditions, which is three times larger than the linewidths of the underlying excitonic and photonic resonances, propelling the system well into the strong coupling regime. Using the versatile resonance control afforded by the BIC concept, our experiments reveal a clear increase of the Rabi splitting with lower asymmetries, which we attribute to a complex interplay between radiative quality factor and mode volume.

In sharp contrast to previous intrinsic coupling approaches based on anapoles^20^ or wires^34^, our results incorporate precise control of the radiative loss channel to achieve tunable coupling strengths and polariton populations.

## Numerical metasurface design

**Fig. 1:**
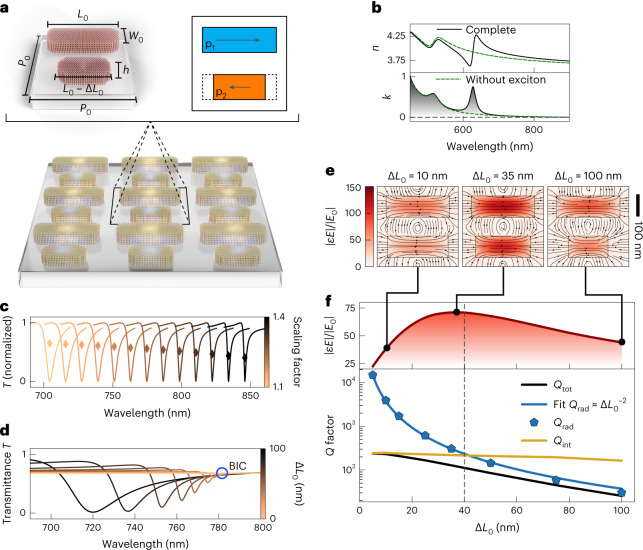
Bound states in the continuum in bulk WS_2_ metasurfaces. **a**, Rod-type symmetry-protected BIC unit cell showing the working principle of opposing electric dipoles (denoted by p_1_ and p_2_). The geometrical unit cell parameters are fixed as: periodicity *P*_0_ = 340 nm, base rod length *L*_0_ = 266 nm and rod width *W*_0_ = 90 nm. The centre points of the rods are placed at the unit cell coordinates (*x*, *y*) = (0, *P*/4) and (0, 3*P*/4). Tunability of the resonance position is realized by introducing a multiplicative scaling factor *S*, which scales the in-plane geometrical parameters according to $P=SP_0$, $L=S{L}_{0}$, $\Delta L=S\Delta {L}_{0}$ and $W=S{W}_{0}$. *h*, height. **b**, Tauc–Lorentz material of WS_2_ showing the real part *n* and imaginary part *k* of the in-plane complex refractive index with and without the exciton. **c**, Simulated transmittance spectra of BIC resonances on the low-energy side of the exciton tuned via scaling of the in-plane geometric parameters. The markers indicate the modulation of the transmittance signal. **d**, Simulated transmittance spectra of BICs for different asymmetries Δ*L*_0_. Smaller asymmetries lead to a spectral redshift and a reduction of the linewidth. For symmetric structures the resonance vanishes as the quasi-BIC turns into a true BIC. **e**,**f**, Electric field enhancements and quality factors for different asymmetry parameters Δ*L*_0_. The maximum field enhancement is achieved when both intrinsic and radiative damping rates of the BIC mode are matched (**e**). The radiative quality factor follows the expected inverse square dependence of a BIC. The intrinsic *Q* factor shows a slight increase for lower asymmetries due to a slightly larger extinction value of the blue-shifted BIC (**f**). The total *Q* factor follows the radiative *Q* factor for large asymmetries and is dominated by the intrinsic *Q* factor for small asymmetries.

WS_2_ was chosen for our BIC metasurface realization because of its spectrally isolated exciton at 629 nm for the bulk material, its large oscillator strength compared with other TMDCs such as MoS_2_, MoSe_2_ or WSe_2_ (ref. ^13^) and its high refractive index (*n* = 4.1 at *λ* = 800 nm). Our design adopts a rod-type BIC unit cell geometry (Fig. 1a) with precise control over the asymmetry via the length difference Δ*L*_0_, spectral tunability via a multiplicative scaling factor *S*, high signal modulation and a spectrally clean mode structure. The WS_2_ metasurfaces were numerically optimized to simultaneously obtain spectral overlap of the BIC resonances with the exciton, strong near-field enhancements and compatibility with nanofabrication tolerances. To capture the optical response of the WS_2_ BIC metasurface with and without the influence of the exciton, we modelled the refractive index of WS_2_ as a Tauc–Lorentz dielectric (Fig. 1b and Methods). We demonstrated the existence of a BIC on the low-energy side of the exciton with full-wave simulations and showed the capability to spectrally tune the BIC via the geometric scaling factor *S* (Fig. 1c), and to control the resonance linewidth with a varying asymmetry parameter Δ*L*_0_ (Fig. 1d). At resonance, the electric near field $\varepsilon E$, where $\varepsilon$ is the permittivity of WS_2_ or air, is concentrated inside the structure (Fig. 1e), leading to an enhancement of the light-matter interaction. To analyse the loss channels associated with the BIC modes, we employed a temporal coupled mode theory model to fit the simulated transmittance spectra. This approach allowed us to decompose the quality factors of the BICs into their radiative and intrinsic parts, where the radiative contribution shows the expected characteristic inverse square law with asymmetry. The intrinsic part incorporates all non-radiative loss channels such as material absorption, surface roughness or edge losses^35^ and is described by our effective material parameters (Methods). Due to small losses in the material, the total *Q* factor is limited by the intrinsic quality factor, which leads to a deviation of the inverse square dependence. Additionally, the BIC-driven light-matter interaction is governed by critical coupling^36^, where the highest field enhancement is achieved when the radiative and intrinsic *Q* factors match (Fig. 1e,f).

## Metasurface fabrication and optical characterization

**Fig. 2:**
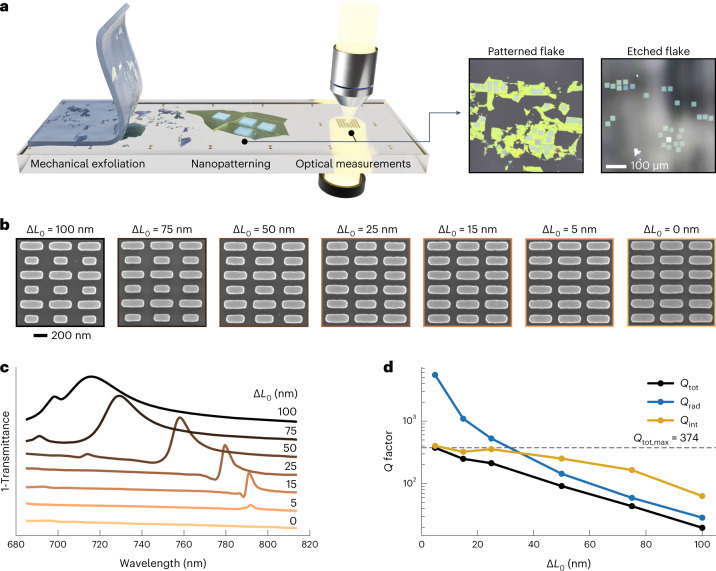
Experimental realization of bulk WS_2_ BIC metasurfaces. **a**, Sketch of the experimental process including exfoliation of WS_2_, nanopatterning via EBL and RIE and optical far-field spectroscopy. **b**, Scanning electron microscope micrographs of fabricated WS_2_ metasurfaces with a scaling factor of *S* = 1.25 with different asymmetry parameters Δ*L*_0_ and a uniform thickness of 80 nm. **c**, Transmittance spectra of WS_2_ metasurfaces (shifted for visiblility) for different Δ*L*_0_, showing the formation of the quasi-BIC modes. To show the uncoupled BIC modes, resonances were placed spectrally separated from the WS_2_ exciton. **d**, Resonance *Q* factors extracted from the spectra in panel **c** showing a maximum *Q* factor of 374.

The fabrication process began with mechanical exfoliation of bulk WS_2_ flakes onto fused silica substrates (Fig. 2a), with flake thicknesses ranging from 30 to 50 nm. The exfoliated WS_2_ flakes were then patterned via EBL and RIE (Methods). Scanning electron microscope micrographs for different asymmetry parameters revealed the accurate reproduction of the metasurface design with high uniformity and surface quality (Fig. 2b). The transmittance spectra of the fabricated structures were measured in an optical spectroscopy setup. We illustrated the emergence of the BIC mode from the symmetric case by gradually increasing the asymmetry parameter Δ*L*_0_. The corresponding transmittance spectra (Fig. 2c) clearly show a spectral blueshift and linewidth broadening with increasing asymmetry and the extracted *Q* factors range from 20 to 370, with highest values found for the lowest asymmetries (Fig. 2d).

As already seen in the simulations, the total *Q* factor is limited by the material-intrinsic absorption as well as scattering losses due to surface roughness and statistical variations^27^. Overall, we see excellent agreement between experimental and simulated results, although the radiative *Q* factor slightly deviates from the characteristic inverse square dependence of the BIC due to small uncertainties in the fits of the experimental spectra for low asymmetries caused by small signal modulations.

## BIC-driven intrinsic strong coupling

**Fig. 3:**
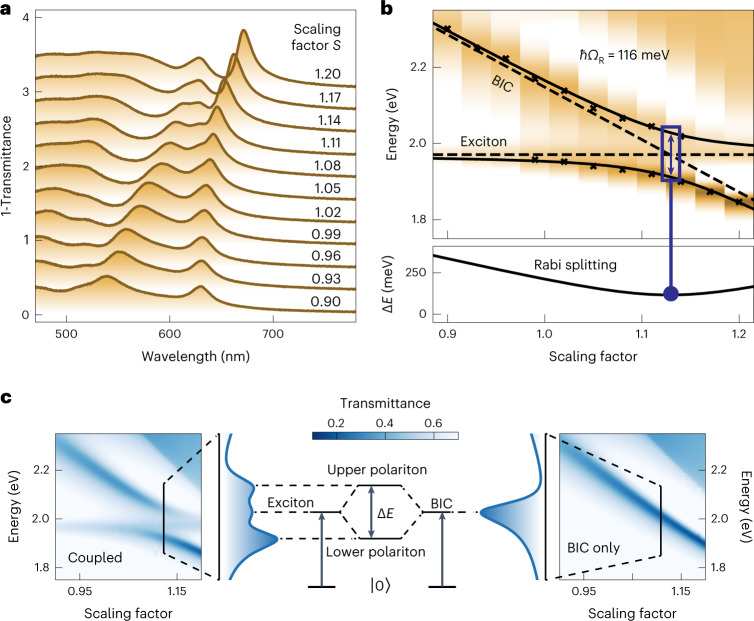
Strong coupling in WS_2_ BIC metasurfaces. **a**, Experimental transmittance spectra of WS_2_ BIC metasurfaces with an asymmetry parameter Δ*L*_0_ = 80 nm for different scaling factors show a characteristic anticrossing mode pattern close to the WS_2_ exciton. **b**, The energy dispersion fit of both polariton branches reveals a Rabi splitting of 116 meV at room temperature. **c**, Simulations where the WS_2_ BIC mode is continuously tuned over the spectral location of the WS_2_ exciton by varying the scaling factor using dielectric functions from the Tauc–Lorentz model with and without the exciton. By aligning the spectral location of BIC and exciton, both modes hybridize into polaritonic branches with higher and lower energies compared with their uncoupled ground states.

To demonstrate metasurface-based strong light-matter coupling, we tuned the BIC resonances over the WS_2_ exciton wavelength at 629 nm (1.971 eV). The spectral tuning was achieved by fabricating 11 metasurface patches with an asymmetry parameter of Δ*L*_0_ = 80 nm with scaling factors between *S* = 0.9 and *S* = 1.2 from a single WS_2_ flake with a height of *h* = 45 nm. Transmittance spectra of the metasurface patches show clear anticrossing behaviour, which is a necessary feature of the strong coupling regime (Fig. 3a). The full energy dispersion of the system is shown in Fig. 3b and is plotted against energy to facilitate the subsequent analysis. The energies of the upper and lower polariton branches can be calculated from

1 $$\begin{array}{l}{\omega }_{\pm }=\frac{{\omega }_{{\rm{BIC}}}+{\omega }_{{\rm{Ex}}}}{2}+\frac{i\left({\gamma }_{{\rm{BIC}}}+{\gamma }_{{\rm{Ex}}}\right)}{2}\\\quad\ \pm \sqrt{{g}^{2}-\frac{1}{4}{\left({\gamma }_{{\rm{BIC}}}-{\gamma }_{{\rm{Ex}}}+i\left({\omega }_{{\rm{BIC}}}-{\omega }_{{\rm{Ex}}}\right)\right)}^{2}},\end{array}$$

where ${\omega }_{{\rm{Ex}}}$ and ${\gamma }_{{\rm{Ex}}}$ indicate the frequency and the exciton damping rate, ${\omega }_{{\rm{BIC}}}$ and ${\gamma }_{{\rm{BIC}}}$ are the resonance frequency and damping rate of the BIC, respectively, $g$ is the coupling strength between the two coupled systems and *i* is the imaginary unit (Supplementary Note 2). The corresponding Rabi splitting is defined as ${\varOmega }_{{\rm{R}}}=\,2\sqrt{{g}^{2}-{\left({\gamma }_{{\rm{BIC}}}-{\gamma }_{{\rm{Ex}}}\right)}^{2}/4}$. The properties of the exciton are extracted from transmission measurements on a symmetric metasurface patch without the presence of a BIC mode, yielding $\hslash {\omega }_{{\rm{Ex}}}=1.971\,{\rm{eV}}$ and $\hslash {\gamma }_{{\rm{Ex}}}=36\,{\rm{meV}}$. The linewidth of the BIC at the wavelength of the exciton is obtained from simulations as $\hslash {\gamma }_{{\rm{BIC}}}=30\,{\rm{meV}}$ (Fig. 3c). We can now fit equation (1) to the experimentally obtained energies of the polariton branches to obtain a Rabi splitting of (116 ± 4) meV. Two established criteria of strong coupling^30,37^

2 $${c}_{1}={\varOmega }_{{\rm{R}}}/({\gamma }_{{\rm{BIC}}}+{\gamma }_{{\rm{Ex}}}) > 1$$

3 $${c}_{2}=g/\sqrt{({{\gamma }_{{\rm{BIC}}}}^{2}+{{\gamma }_{{\rm{Ex}}}}^{2})/2} > 1$$

yield ${c}_{1}=1.75$ and ${c}_{2}=1.74$, respectively, exceeding the required conditions substantially and verifying that our BIC-driven metasurface concept indeed reaches the strong coupling regime. These findings are further supported by analysing the influence of potential uncertainties in our fitting scheme (Supplementary Fig. 5).

Furthermore, we investigated the influence of temperature on the coupling process by measuring the transmittance spectra of the WS_2_ BIC metasurface at different temperatures in a cryostat. The optical performance of the metasurface is maintained even at cryogenic temperatures, and comparing the experiments at room temperature and 5 K, the expected spectral blueshift of the exciton^38^ is visible (Supplementary Fig. 7a). Although the Rabi splitting shows a small declining trend with decreasing temperatures (Supplementary Fig. 7b), which we ascribe to the reduction of the oscillator strength for the phonon-mediated momentum indirect transition of the bright exciton, strong coupling is maintained down to 5 K.

## Tailored strong coupling

**Fig. 4:**
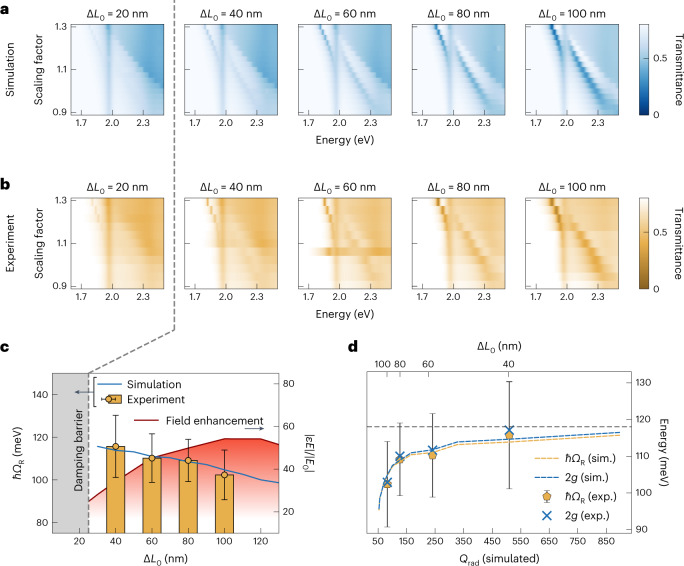
Tailored Rabi splitting in BIC metasurfaces. **a**,**b**, Simulated (**a**) and experimental (**b**) energy dispersion plots for WS_2_ BIC metasurfaces for different asymmetry factors Δ*L*_0_, enabling strong coupling experiments with multiple resonance quality factors and field confinements. **c**, Extracted Rabi splitting values from the data in **a** and averaged electric field enhancement in the resonator as a function of asymmetry parameter Δ*L*_0_. The Rabi splitting decreases with increasing asymmetry, demonstrating the capability of tailouring light-matter coupling strength using photonic BICs. **d**, Rabi splitting values and coupling constants extracted from **b** as a function of the simulated radiative BIC quality factor saturating for high radiative quality factors. The error bars depict the standard error of the fit values. Exp., experimental; sim., simulated.

To correlate BIC quality factor and Rabi splitting, we simulated and fabricated metasurfaces with different asymmetry factors Δ*L*_0_ with a height of 33 nm. For higher asymmetries, the BIC resonances exhibit higher modulation contrasts combined with broader linewidths (Fig. 4a,b). On the other hand, we observe increased values of the Rabi splitting for lower asymmetries (Fig. 4c). This unique and previously unavailable tuning behaviour provides a new mechanism for tailoring the Rabi splitting using simple structural modification, and can thus enable the design of metasurface geometries with optimal ratio of resonance modulation to Rabi splitting in the strong coupling regime (Supplementary Fig. 6). For the lowest asymmetry of Δ*L*_0_ = 20 nm, the BIC resonances are strongly damped by the intrinsic losses of WS_2_, and we can no longer resolve a clear peak splitting (grey shaded area in Fig. 4c).

Furthermore, we plotted the Rabi splitting against the radiative *Q* factors of the BIC resonances without exciton from simulations and observed that both the Rabi splitting and the coupling constant *g* undergo a steep increase at low *Q* factors and saturate at higher *Q* factors (Fig. 4d). The observed trend is consistent with predictions from the literature, where the saturation of the Rabi splitting is attributed to the saturation of the electric field enhancement^39,40^. However, in comparison with idealized lossless dielectric BIC-driven metasurfaces, where the field enhancement scales with the square root of the *Q* factor (Supplementary Fig. 9i) and thus shows a saturating behaviour, the field enhancement of realistically modelled WS_2_ BIC metasurfaces is governed by critical coupling, which is influenced by background losses in the material. At the spectral position of the exciton, maximum field enhancement is achieved for Δ*L*_0_ = 110 nm (Fig. 4c) and shows an upward trend, which contrasts strongly with the downward trend of the Rabi splitting, implying that the Rabi splitting is independent of the electric near-field enhancement. This is also supported by additional simulations with lossless and lossy materials, showing identical Rabi splitting (Supplementary Fig. 9c,f).

The observed independence from the field enhancement is consistent with the common definition for the coupling strength *g*^41^

4 $$g \approx {{\mu }}\cdot {{E}} \approx \sqrt{\frac{N}{{V}_{{\rm{eff}}}}},$$

where *µ* is the collective dipole moment of the exciton, *E* the local electric field strength, *N* the number of excitons participating in the coupling process and *V*_eff_ the effective mode volume. We calculated the effective mode volume, which follows the decreasing trend of the geometric volume, when increasing the asymmetry (Supplementary Note 5 and Supplementary Fig. 11), which would imply an increase of coupling strength. However, due to the reduced volume, fewer excitons partake in the coupling process, which effectively counteracts the effects of the reduction of mode volume, leading to constant exciton density and coupling strength. Notably, the above definition of the coupling strength is only valid for low-loss and non-radiative cavities^42^. A similar trend is observed for the optical confinement factor of the BIC structure (Supplementary Fig. 12).

Next, we separately studied the radiative and intrinsic loss channels that affect the total BIC *Q* factor. We used a simplified Lorentzian material representing a narrower exciton with a linewidth that is between the narrowest and the broadest BIC linewidths achievable with the given geometric parameters. When we change the *Q* factor by adding intrinsic losses to the system, we observe a constant coupling strength *g* and a Rabi splitting peaking at the maximum value 2 *g* for matching linewidths of BIC and exciton (Supplementary Fig. 8a,b). The decrease of Rabi splitting for low *Q* factors is caused by the expected weakening of the strong coupling conditions towards the weak coupling regime (Supplementary Fig. 8c).

In contrast, when changing radiative losses through a variation of the asymmetry parameter Δ*L*_0_, both the coupling strength and Rabi splitting follow the saturation trend described above, independent of intrinsic losses of the system, and the Rabi splitting reaches the value 2 *g* when the linewidths of BIC and exciton match (Supplementary Fig. 9j). This shows that the general coupling rules are still valid, but that these effects are negligible compared with the overall trend. Importantly, the coupling strength itself is modified by varying the radiative *Q* factor, showing that our TMDC BIC platform is able to directly control the coupling conditions in the system.

Therefore, we deduced that the coupling strength (Supplementary Fig. 9c,f) is indeed independent of the electric near-field enhancement (Supplementary Fig. 9d,g). Moreover, considering the constant excitonic density, our analysis identified an additional mechanism for changing the coupling strength by controlling the radiative loss channel. While intrinsic losses change the Rabi splitting because of a linewidth broadening of the BIC, eventually turning to the weak coupling regime, energy is dissipated from the resonator system through the radiative loss channel to the far field, which reduces the interaction time of the photons with the excitons for large asymmetry parameters, thus lowering the coupling strength.

Even though the coupling strength itself is independent of any field-enhancing effect such as critical coupling, the population of the polariton branches is heavily influenced by it. When the radiative BIC quality factor is matched to both its intrinsic quality factor and the width of the exciton, the maximum absorbance for our system of 0.5 can be reached, which is known as strong critical coupling or polaritonic critical coupling^31^ (Supplementary Fig. 10a–c). We numerically proved that this regime can be reached for our Tauc–Lorentz material with only small changes to the structure parameters of the BIC unit cell (Supplementary Fig. 10d).

## Discussion

We have realized a self-hybridized WS_2_ metasurface platform based on symmetry-protected BICs. We achieved resonances with total *Q* factors of up to 370 on the low-energy side of the exciton at wavelengths from 700 to 800 nm, demonstrating that exfoliated bulk TMDCs are an excellent material for all-dielectric nanophotonics owing to their high refractive index, low losses and mono-crystallinity. Moreover, we leveraged the precise control of the resonance position of the BIC mode via geometric variations to tune the BIC spectrally to the material-intrinsic excitonic resonance at 629 nm, leading to strong light-matter interaction and hybridization of photonic and excitonic modes. We experimentally resolved a clear anticrossing pattern of the exciton-polariton branches with a Rabi splitting of 116 meV at temperatures ranging from 5 K to room temperature. By varying the radiative *Q* factor of the BIC via the asymmetry factor of the BIC unit cell between Δ*L*_0_ = 40 nm and Δ*L*_0_ = 100 nm, we were able to tune the Rabi splitting in a linear fashion, where the highest Rabi splitting value was reached for the largest *Q* factors. We have further shown that the Rabi splitting is independent of the electric near-field enhancement, rendering the coupling conditions of our TMDC BIC platform independent of the material-intrinsic losses. Furthermore, because of the precise control over the radiative coupling conditions of the BIC to the far field, we can tune the coupling strength directly, which is facilitated through a complex interplay between mode volume and temporal field confinement via the radiative loss channel.

In comparison with other strong coupling platforms such as anapole disks, bulk wires or hybrid plasmonic system, we achieved direct control over the coupling strength via tuning the radiative quality factor via the geometrical asymmetry factor. Moreover, our numerical simulations showed that we can maximize the population of the polariton branches by reaching the polaritonic critical coupling regime, which could be crucial for the development of efficient polaritonic devices.

Recent advances in the growth of high-quality bulk TMDC films via chemical vapour deposition^43^ show a way forward to area-scalable TMDC BIC metasurfaces unrestricted by the size limitations of exfoliated crystals.

Through our generalized description of WS_2_ as a Lorentz-type dielectric, our results are broadly applicable to other materials systems supporting resonant material-intrinsic responses such as perovskites or other van der Waals materials like black phosphorus and hexagonal boron nitride, which could pave the way for robust, tunable and efficient polaritonic devices in a large wavelength region.

## Methods

### Numerical simulation

Simulations were conducted with CST Studio Suite 2021 using periodic Floquet boundary conditions and a plane-wave excitation polarized along the long axis of the rods with normal incidence angle. The WS_2_ material data used throughout the paper were adapted from tabulated permittivity data for monolayer WS_2_ from ref. ^44^ by fitting a Tauc–Lorentz model with four oscillators to represent the in-plane permittivity. We accounted for the spectral shift of the exciton and a reduction of oscillator strength for the bulk material by changing the corresponding values of the material and confirmed the correctness of simulation by comparing with results from optical characterization. This method allows a combination of a multitude of non-radiative loss channels, such as material absorption, surface scattering and the effect of nanostructuring on the exciton into an empirical permittivity model, yielding excellent experimental agreement while reducing computational complexity. The exact formulas and parameters can be found in Supporting Note 1. The out-of-plane permittivity was set to *ε* = 7 + 0i.

### Nanofabrication

WS_2_ flakes were transferred from bulk crystals (HQ Graphene) onto fused silica substrates via mechanical exfoliation. By controlling exfoliation parameters, such as the heat treatment temperature, exfoliation time and applied pressure, we optimized the yield of the flakes with thicknesses ranging from 30 to 50 nm to achieve an adequate area to host a multitude of BIC metasurfaces. The metasurface patches have a footprint of 35 × 35 μm^2^, which ensures a sufficient number of more than 50 × 50 unit cells for each scaling factor—a necessity to excite the collective BIC resonance with stable *Q* factor^45^. The substrates were treated with oxygen plasma to enhance the adhesion and the deposition took place at elevated temperatures of above 100 °C to remove moisture and to stretch out the tape used for exfoliation, which subsequently flattened the TMDC flakes to avoid shattering into small shards. Small mechanical pressure was applied during deposition. For alignment purposes, a marker system was fabricated onto the substrates via optical lithography (SÜSS Maskaligner MA6) before exfoliation. The height of the flakes was measured with a profilometer (Bruker Dektak XT) using a stylus with a radius of 2 µm, providing sub-nanometre resolution. A layer of PMMA 950k resist followed by Espacer 300Z was spin-coated onto the sample, after which the metasurface design was written into the resist via EBL (Raith ELine Plus). A gold hardmask was deposited with e-beam evaporation followed by a lift-off in Microposit Remover 1165. Finally, the design was etched into the flakes using RIE (Oxford PlasmaPro 100) with a SF_6_-based chemistry at a pressure of 20 mTorr and a radio frequency (RF) power of 50 W. The hardmask was removed in a solution of potassium monoiodide and iodine (Sigma-Aldrich).

### Optical characterization

The samples were characterized in a confocal optical transmission microscope with from-the-bottom illumination with collimated and linear polarized white light. The light was collected using a ×50 objective with a numerical aperture of 0.8, dispersed in a spectrometer with a grating groove density of 150 mm^−1^ and detected with a SI-CCD sensor.

The low-temperature transmittance measurements were performed in a variable-temperature helium flow cryostat with a confocal microscope in transmission configuration. Thermal light from a tungsten halogen light source was used for excitation and was focused from-the-bottom into the sample. The light transmitted through the sample was collected using a ×20 objective with a numerical aperture of 0.4. A pinhole was used as a spatial filter in the detection path yielding a detection spot size of around 7 μm. The large footprints of the metasurfaces were leveraged to collect light only in the centre of the arrays, which mitigates edge effects and allows for optimal signal modulations. The collected light was analysed in a 500 mm spectrometer using a 150 grooves per mm grating and detected using a CCD sensor.

### Reporting summary

Further information on research design is available in the Nature Portfolio Reporting Summary linked to this article.

## Online content

Any methods, additional references, Nature Portfolio reporting summaries, source data, extended data, supplementary information, acknowledgements, peer review information; details of author contributions and competing interests; and statements of data and code availability are available at 10.1038/s41563-023-01580-7.

## Supplementary information

Supplementary Information Supplementary Figs. 1–12, Tables 1 and 2, and Discussion.

Reporting Summary

## Data Availability

The main data supporting the findings of this study are available within the article and its Supplementary Information files. Extra data are available from the corresponding author upon reasonable request.
