# Supplementary material for: Intrinsic strong light-matter coupling with self-hybridized bound states in the continuum in van der Waals metasurfaces
Source: Nat Mater. 2023 Jun 22;22(8):970–6. doi: 10.1038/s41563-023-01580-7 (PMC10390334; doi:10.1038/s41563-023-01580-7)
Supplement: Supplementary file 1 — Supplementary Figs. 1–12, Tables 1 and 2, and Discussion. [file 41563_2023_1580_MOESM1_ESM.pdf]

nature materials

Article

https://doi.org/10.1038/s41563-023-01580-7

# Intrinsic strong light-matter coupling with self-hybridized bound states in the continuum in van der Waals metasurfaces

In the format provided by the authors and unedited

**Table of Contents**


Table of Contents ........ 1

Supplementary Note 1: Tauc-Lorentz material model for $WS_2$ ........ 2

Table S1: Parameters for artificial WS2 Tauc-Lorentz material. ........ 2

Figure S1: Tauc-Lorentz fits of monolayer WS2. ........ 3

Figure S2: Comparison between tabulated and adapted bulk model. ........ 3

Supplementary Note 2: Complete and simplified coupled oscillator models for strong coupling ........ 3

Figure S3: Sketch of the used TCMT model with two ports and three resonances ........ 4

Figure S4: Comparison between TCMT and dispersion fit for strong coupling. ........ 6

Figure S5: Influence of uncertainties of resonance linewidths on strong coupling. ........ 6

Figure S6: Strong-coupling criteria for different BIC asymmetry parameters. ........ 7

Figure S7: Experimental results of measurements in a cryostat. ........ 7

Supplementary Note 3: Effect of the radiative and intrinsic quality factors on the coupling strength and Rabi splitting ........ 8

Figure S8: Effect of the intrinsic quality factor on the coupling strength and Rabi splitting. ........ 9

Figure S9: Effect of radiative quality factor on coupling strength and Rabi splitting. ........ 10

Supplementary Note 4: Polaritonic critical coupling ........ 11

Figure S10: Polaritonic critical coupling. ........ 13

Figure S11: Effective mode volumes for TMDC BICs. ........ 14

Figure S12: Optical confinement factor for different imaginary parts of the permittivity. ........ 16

Table S2: Comparison with other strong-coupling implementations ........ 16

References ........ 17

**Supplementary Note 1: Tauc-Lorentz material model for $WS_2$**

The imaginary part of the permittivity of a Tauc-Lorentz material is given by

$$\mathrm{Im}\,\varepsilon(E) = \begin{cases} \dfrac{A_k E_{0,k} C_k \left(E - E_g\right)^2}{\left(E^2 - E_{0,k}^2\right)^2 + C_k^2 E^2} \dfrac{1}{E}, & \text{for } E > E_g \\ 0, & \text{for } E \leq E_g \end{cases} \qquad \text{(S1)}$$

where $A_k$ is the amplitude (or oscillator strength), $E_{0,k}$ the resonance position and $C_k$ the damping rate of the k-th oscillator and $E_g$ a global bandgap applied to all oscillators set to 300 THz. The real part of the permittivity was retrieved via the Kramers-Kronig relations

$$\mathrm{Re}\,\varepsilon(E) = D + \frac{2}{\pi} P \int_{E_g}^{\infty} \frac{\xi\, Im\left(\varepsilon(\xi)\right)}{\xi^2 - E^2} \mathrm{d}\xi \qquad \text{(S2)}$$

where the background permittivity was set with D to 10.

| k | $A_k$ (arb. u.) | $E_{0,k}$ (THz) | $C_k$ (THz) |
|---|---|---|---|
| 1 (Exciton) | 546.9 | 476.6 | 14.0 |
| 2 | 420.5 | 578.1 | 41.1 |
| 3 | 515.4 | 683.5 | 43.1 |
| 4 | 9091.1 | 735.3 | 216.3 |

**Table S1: Parameters for artificial $WS_2$ Tauc-Lorentz material.**

As described in the methods section of the main text, we fit the above Tauc-Lorentz model to the tabulated material data for monolayer $WS_2$ taken from[1] with good agreement (Fig. S1). The model was then adapted to represent our nanostructured bulk material via optical measurements, resulting in the parameters in Table S1. As can be seen from Figure S2, the exciton undergoes a spectral redshift and a reduction of its oscillator strength.

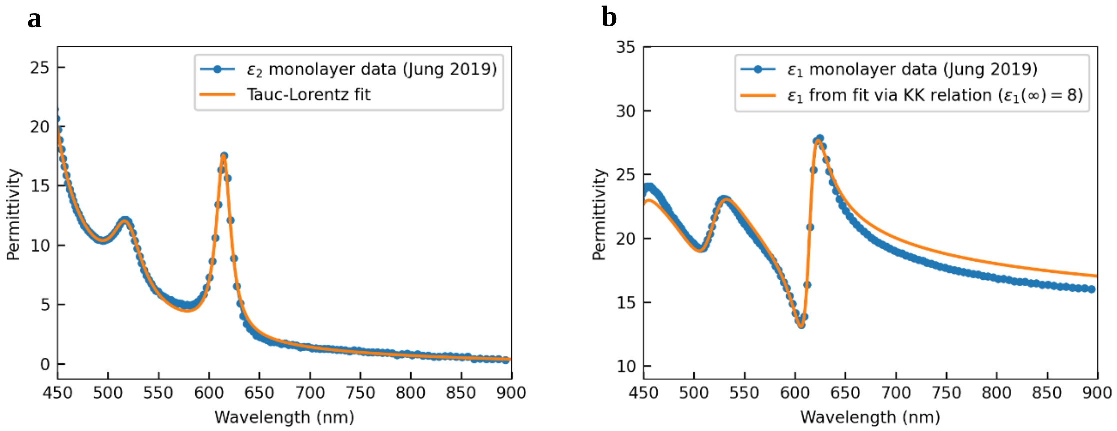


**Figure S1: Tauc-Lorentz fits of monolayer $WS_2$. a**, Tauc-Lorentz model fit to tabulated imaginary part of the in-plane $WS_2$ permittivity from[1]. **b**, Tauc-Lorentz model fit with parameters taken from panel (a), fit to the real part of the $WS_2$ permittivity via a constant offset.

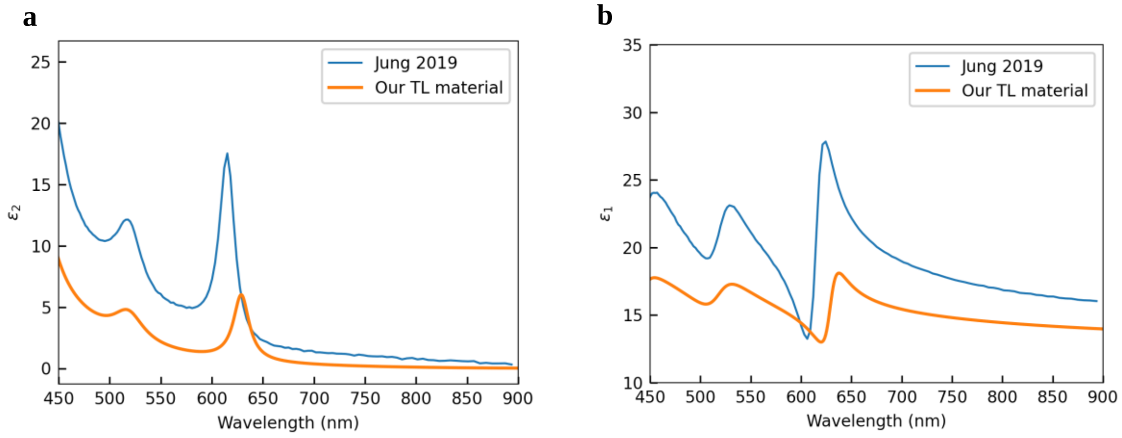


**Figure S2: Comparison between tabulated and adapted bulk model. a**, Comparison between the imaginary parts of the in-plane $WS_2$ permittivity for monolayers and our adapted bulk model. **b**, Comparison from panel (a) for the real parts of the permittivity.

### Supplementary Note 2: Complete and simplified coupled oscillator models for strong coupling

A powerful toolkit to describe resonances in open cavities and their coupling properties to both the far field and to other modes is the temporal coupled mode theory (TCMT)[2].

Here, we utilize a model consisting of ports, which allows for transmitted and reflected waves, to accurately describe the conditions of our TMDC BIC metasurface (**Figure S1**). The system is excited via external fields through port 1 ($\mathbf{s}_+ = (s_{1+}, 0)^\mathrm{T}$), which are subsequently affected by the metasurface,

described by the scattering matrix S, and decay through port 1 and 2 with equal proportion due to mirror symmetry ($\mathbf{s}_- = (s_{1-}, s_{2-})^{\mathrm{T}}$) (Equation (S1)).

$$\mathbf{s}_- = S\mathbf{s}_+ \quad \text{(S3)}$$

For the two-port system the scattering matrix S is a symmetric 2x2 matrix given by

$$S = \begin{pmatrix} s_{11} & s_{12} \\ s_{21} & s_{22} \end{pmatrix} = C + K[\mathrm{i}(\omega I - \Omega) + \Gamma]^{-1} K^T, \quad \text{(S4)}$$

where we can attribute the S-parameter $s_{11} = \frac{s_{1-}}{s_{1+}} = r$ with the reflection and $s_{21} = \frac{s_{2-}}{s_{1+}} = t$ with the transmission coefficient. The resonance frequencies and intrinsic damping rates of the modes of the cavity are given by $\Omega$ and the radiative damping rates by $\Gamma$. The port-cavity coupling is described by $K$, which is a function of the radiative damping rates. The direct port-port crosstalk by the unitary matrix $C$.

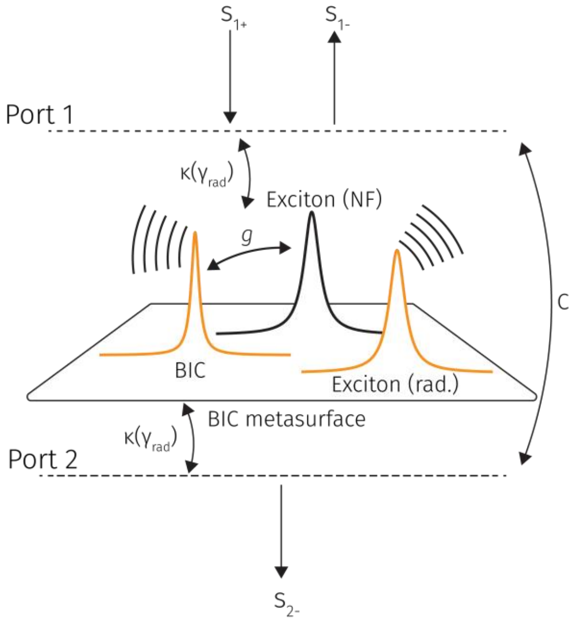


**Figure S3: Sketch of the used TCMT model with two ports and three resonances**. BIC and two excitons to describe the near-field coupling and radiative contributions. The metasurface is illuminated from above ($s_{1+}$) and allows for reflected ($s_{1-}$) and transmitted waves ($s_{2-}$). The near-field coupling between BIC and the exciton is described by the coupling parameter g, the far-field coupling via the parameter κ as a function of the radiative damping rate of the respective resonance. Direct port coupling is addressed via parameter C.

From experimental and simulated transmittance spectra with strong-coupling fingerprint, a distinct three-dip feature is visible, consisting of the coupled BIC-exciton polariton and radiative parts of the exciton itself, which do not participate in the coupling process. Thus, we can describe the system effectively by a cavity supporting three resonant modes (BIC and a two-fold contribution of the exciton), where one part only interacts with the BIC in the near-field via the coupling strength $g$ and the other part only via the radiative far-field via the term $\sqrt{\gamma_{\text{BIC,rad}}\,\gamma_{\text{Ex,rad}}}$. The complete set of parameters is given by the following:

$$\Omega = \begin{pmatrix} \omega_{\text{BIC}} + \mathrm{i}\gamma_{\text{BIC,int}} & g & 0 \\ g & \omega_{\text{Ex}} + \mathrm{i}\gamma_{\text{Ex,int}} & 0 \\ 0 & 0 & \omega_{\text{Ex}} + \mathrm{i}\gamma_{\text{Ex,int}} \end{pmatrix}$$

$$\Gamma = \begin{pmatrix} \gamma_{\text{BIC,rad}} & 0 & \sqrt{\gamma_{\text{BIC,rad}}\,\gamma_{\text{Ex,rad}}} \\ 0 & 0 & 0 \\ \sqrt{\gamma_{\text{BIC,rad}}\,\gamma_{\text{Ex,rad}}} & 0 & \gamma_{\text{Ex,rad}} \end{pmatrix}$$

$$K = \begin{pmatrix} \sqrt{\gamma_{\text{BIC,rad}}} & 0 & \sqrt{\gamma_{\text{Ex,rad}}} \\ \sqrt{\gamma_{\text{BIC,rad}}} & 0 & \sqrt{\gamma_{\text{Ex,rad}}} \end{pmatrix}$$

$$C = e^{i\phi} \begin{pmatrix} r_0 & \mathrm{i}t_0 \\ \mathrm{i}t_0 & r_0 \end{pmatrix} \tag{S5}$$

This allows to extract the coupling strength using a single spectrum. However, it is limited to simplified materials with Lorentz-type oscillators, that is, no dispersive losses due to inter-band absorption. However, we can reduce the TCMT model to a simpler eigenvalue problem, which we use to fit the dispersion of the polariton branches in the main text. By combining the radiative and intrinsic damping rates into a total damping rate $\gamma_i = \gamma_{i,\text{rad}} + \gamma_{i,\text{int}}$, we arrive at the expression

$$\begin{pmatrix} \omega_{\text{BIC}} + \mathrm{i}\gamma_{\text{BIC}} & g \\ g & \omega_{\text{Ex}} + \mathrm{i}\gamma_{\text{Ex}} \end{pmatrix} \mathbf{v} = \omega_{\pm}\mathbf{v}, \tag{S6}$$

where $\omega_{\pm}$ are the resonance positions of the upper and lower polariton branches and $\mathbf{v}$ contains the Hopfield coefficients, which describe the proportions of BIC and exciton in the hybrid mode[3]. By solving the Eigenvalue problem, the well-known polariton dispersion relation

$$\omega_{\pm} = \frac{\omega_{\mathrm{BIC}} + \omega_{\mathrm{Ex}}}{2} + \frac{i(\gamma_{\mathrm{BIC}} + \gamma_{\mathrm{Ex}})}{2} \pm \sqrt{g^2 - \frac{1}{4}(\gamma_{\mathrm{BIC}} - \gamma_{\mathrm{Ex}} + i(\omega_{\mathrm{BIC}} - \omega_{\mathrm{Ex}}))^2} \quad \text{(S7)}$$

is retrieved, which we use to fit the coupling strength $g$ from experimental results. The Rabi splitting $\Omega_{\mathrm{R}}$ is defined as the minimal polariton splitting $\Delta\omega = \omega_{+} - \omega_{-}$, i.e. when $\omega_{\mathrm{BIC}} = \omega_{\mathrm{Ex}}$, yielding

$$\Omega_{\mathrm{R}} = 2\sqrt{g^2 - (\gamma_{\mathrm{BIC}} - \gamma_{\mathrm{Ex}})^2/4}. \quad \text{(S8)}$$

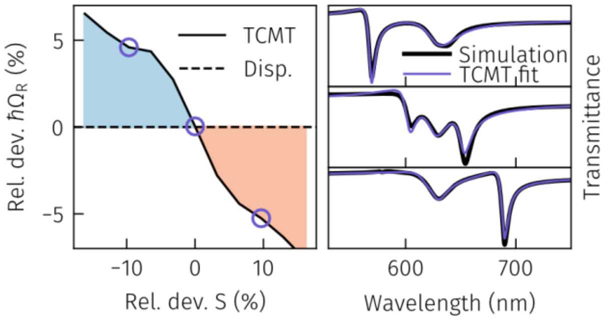


**Figure S4: Comparison between TCMT and dispersion fit for strong coupling.** The Rabi splitting extracted from the TCMT model reveals an increase of coupling strength with a reduction of the unit cell scaling factor and vice versa. The Rabi splitting extracted from the polariton dispersion model is inherently independent of the scaling factor. The comparison of the two models shows that the dispersion model yields the averaged Rabi splitting of all scaling factors and is hence a suitable model to describe strong coupling in $WS_2$ BIC metasurfaces.

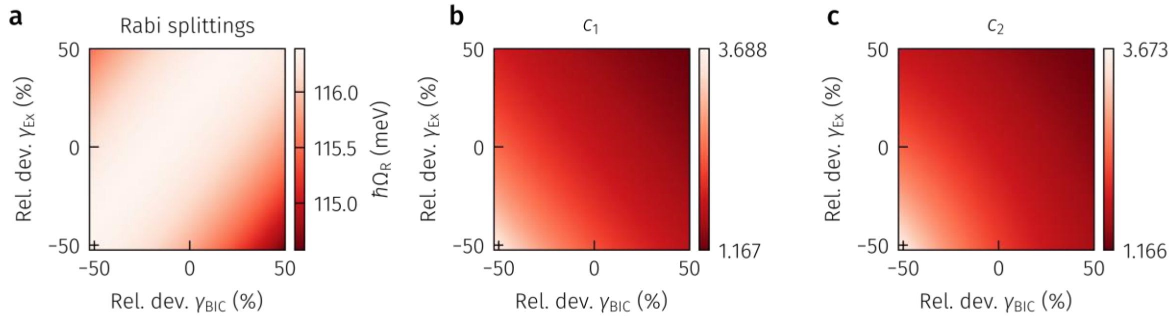


**Figure S5: Influence of uncertainties of resonance linewidths on strong coupling. a**, Fitted Rabi splittings for the data in Figure 3b of the main text for deviating linewidths of BIC and exciton. For the largest mismatch of linewidth (50% narrower exciton and 50% broader BIC), the reduction of the Rabi splitting is less than 2%, which is in the range of uncertainty of the fitted Rabi splitting for $\hbar\gamma_{\mathrm{BIC}} =$ 30 meV and $\hbar\gamma_{Ex} = 36$ meV. **b**, **c**, The strong-coupling criteria $c_1$ and $c_2$ (equations (2) and (3) of the

main text) for the conditions of panel (a) show always values greater than one, proving that the TMDC BIC metasurface reaches a stable point in the strong coupling regime.

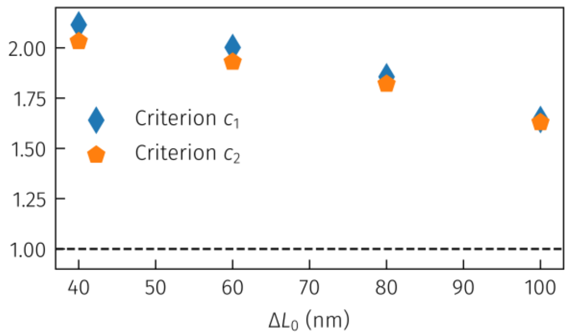


**Figure S6: Strong-coupling criteria for different BIC asymmetry parameters.** Both criteria remain well over the threshold of one, even for largest asymmetry, which shows that the system is always in the strong-coupling regime.

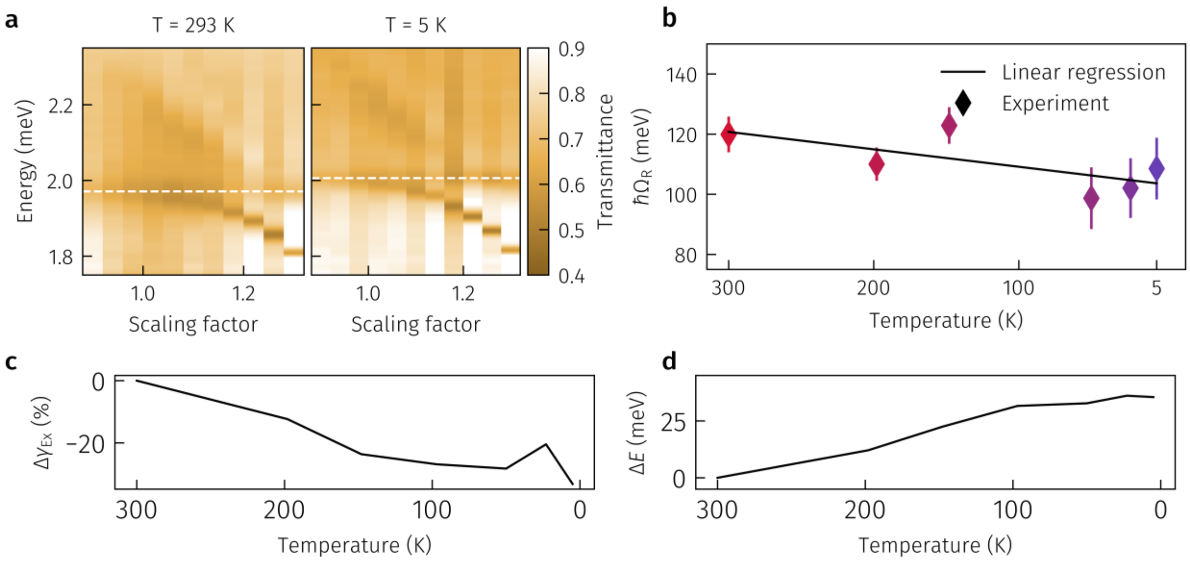


**Figure S7: Experimental results of measurements in a cryostat. a**, Energy dispersion plots of strong coupling in $WS_2$ BIC metasurfaces with an asymmetry parameter $\Delta L_0 = 80$ nm and a height of 33 nm at room temperature ($T$ = 293 K) and cryogenic temperatures ($T$ = 5 K) depicting a spectral blueshift of the $WS_2$ exciton. **b**, Reduced Rabi splitting for low temperatures. The error bars depict the standard error of the fit values. As can be seen in panel (a), the signal strength of the transmission measurements is lower in the cryostat setup, which reduces the accuracy of the strong coupling fit and leads to outliers in the otherwise monotonically decreasing trend of the Rabi splitting.

**c**, Relative change of the excitonic linewidth, extracted from $WS_2$ metasurfaces with symmetric unit cell, showing a decrease for lower temperatures. **d**, Relative change of the energy of the $WS_2$ exciton, showing a spectral blueshift for lower temperatures.

**Supplementary Note 3: Effect of the radiative and intrinsic quality factors on the coupling strength and Rabi splitting**

Our $WS_2$ metasurfaces give great control over the radiative quality factor of the BIC resonances. However, the intrinsic quality factor, which is affected by material-intrinsic losses and other parasitic loss channels, such as fabrication imperfections, sets an upper bound on the total achievable quality factor, especially at low asymmetry parameters $\Delta L_0$ (Figure 2f and 3d). Furthermore, the presence of losses limits and shifts the field enhancement to the critical coupling condition. Here, we compare the effect of both the radiative BIC quality factor (**Figure S6**) by changing the symmetry parameter and the intrinsic BIC quality factor (**Figure S7**). This is achieved by artificially increasing the imaginary part of the permittivity and show that the radiative quality factor directly influences the coupling strength (and thus the Rabi splitting), whereas the intrinsic quality factor has no effect on the coupling strength and only influences the Rabi splitting by means of mismatch of the quality factors of BIC and excitonic response. Moreover, this implies that the field enhancement is not the driving force behind the strong-coupling process.

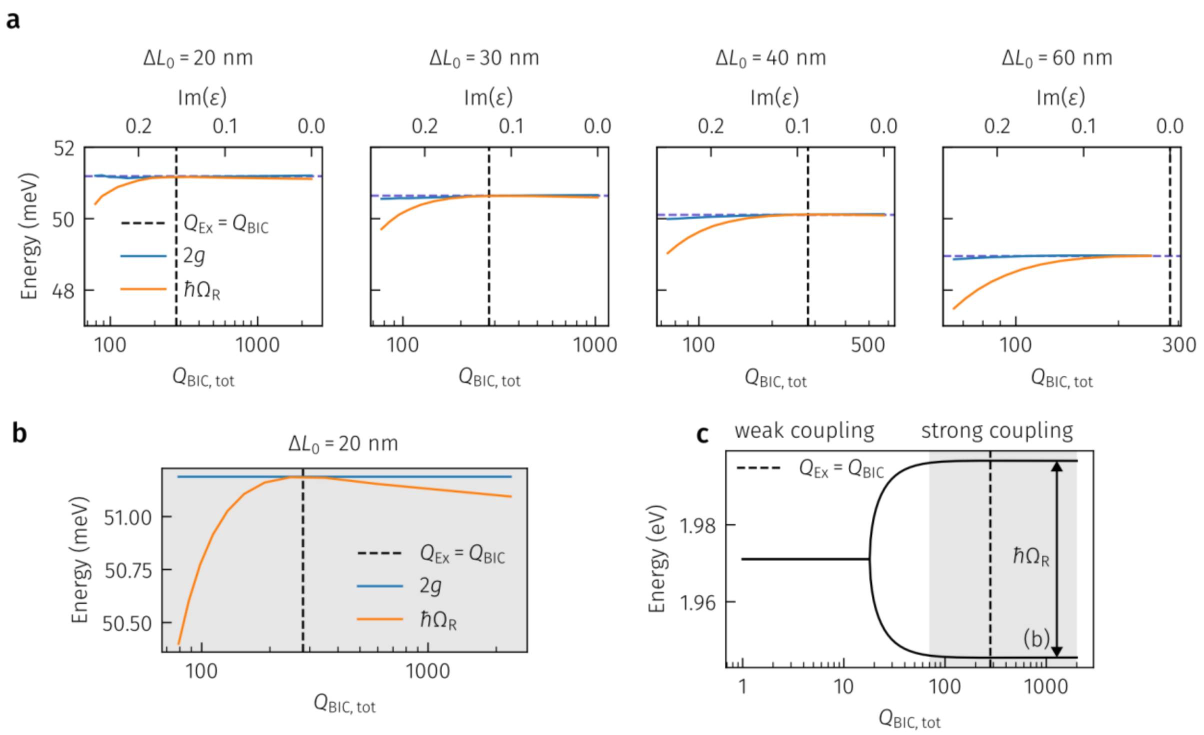


**Figure S8: Effect of the intrinsic quality factor on the coupling strength and Rabi splitting. a**, Fitted Rabi splittings and coupling strengths plotted against the total quality factor, which is varied via intrinsic loss channels (cmp. Figure S2a). The coupling strength is constant over the complete range of quality factors, whereas the Rabi splitting decreases for lower Q factors. Overall, the coupling strength and Rabi splitting decreases for larger asymmetry factors, which is consistent with Figure 2. **b**, Fitted Rabi splittings and coupling strength by assuming the coupling strength is constant for $\Delta L_0$ = 20 nm. The Rabi splitting has a maximum, when the quality factor of the BIC matches the quality factor of the exciton. **c**, Mode splitting for parameters extracted from panel (b). The decrease in Rabi splitting for lower Q factors is caused by moving towards the weak coupling regime.

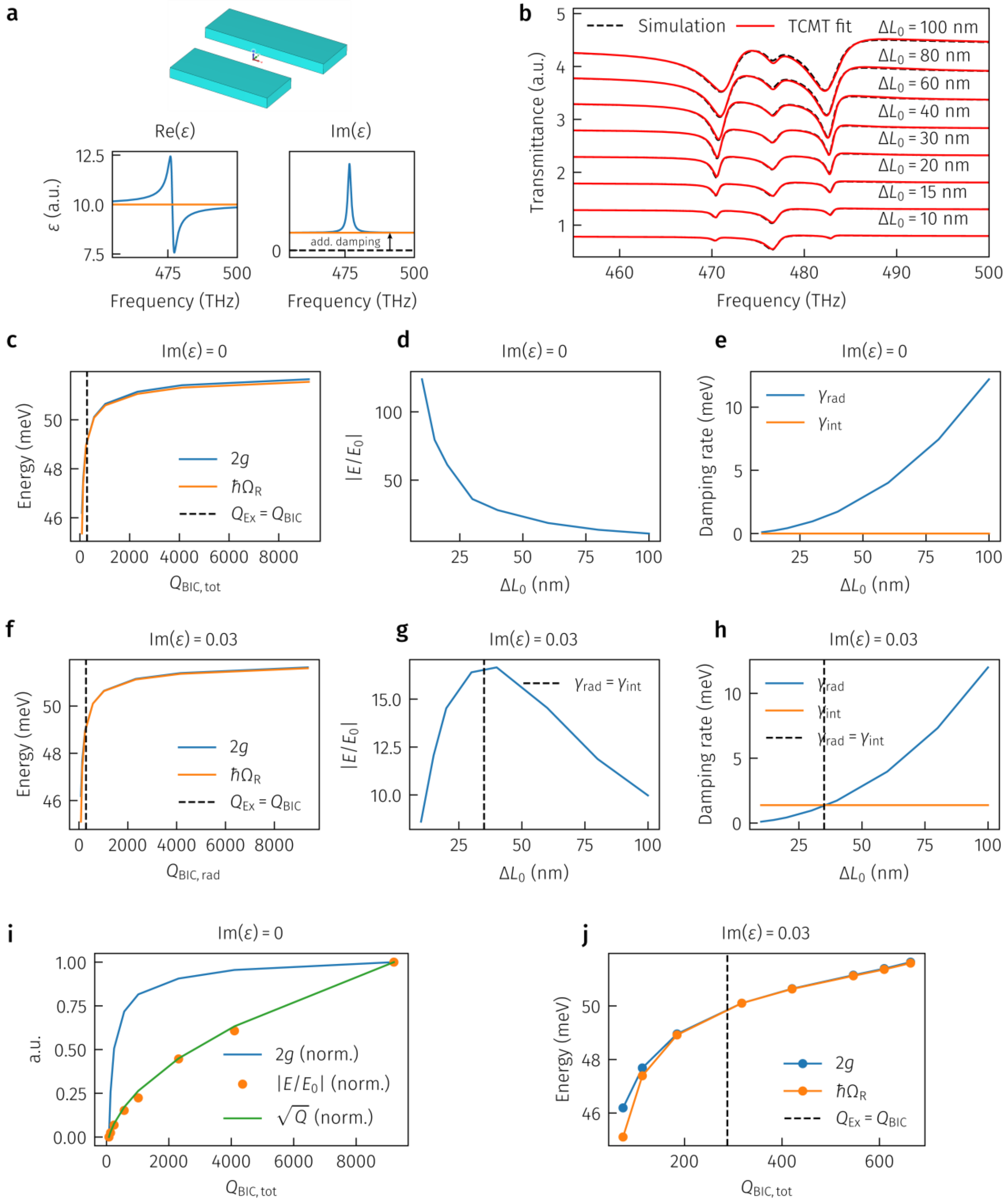


**Figure S9: Effect of radiative quality factor on coupling strength and Rabi splitting. a**, Sketch of a simplified unit cell in vacuum with a height of 33 nm. The lateral geometric parameters are taken from the main text. A simplified Lorentzian material with the constants A = 6.837318, $E_0$ = 476.6 THz, C = 1.4033 THz, $E_g$ = 0 was used, where additional absorption can be introduced via an offset in the

imaginary part of the permittivity. **b**, Simulated and fitted transmittance spectra for different asymmetry parameters. The total excitonic linewidth comprising of the radiative and intrinsic parts extracted from transmittance spectra was fitted to be 0.83011 THz, yielding a Q factor $Q_{Ex}$ = 287.175612, which is without loss of generality chosen to be narrower than the $WS_2$ A-exciton, to be able to resolve features even for high asymmetry factors. **c**, The coupling strength and Rabi splitting plotted against the BIC quality factor for the aforementioned Lorentzian material without additional losses, showing only slight deviation for $Q_{BIC} >> Q_{Ex}$. **d**, Mean field enhancement inside the resonator structure, which scales with different asymmetry parameters as $\Delta L_0^{-1}$. **e**, Intrinsic and external damping rates of the BIC without excitonic response. The radiative rate scales according to the inverse square dependence of the radiative quality factor like $\Delta L_0^2$, whereas the intrinsic damping rate is zero due to vanishing losses. **f-h**, Similar to panels (c) **-** (e) with intrinsic losses introduced via an offset of the imaginary part of the permittivity of 0.03. The coupling strength and Rabi splitting is within the simulation and fit uncertainties identical to the lossless case when assuming the radiative quality factor, whereas the field enhancement shows a different picture. The field enhancement is governed by critical coupling, showing a maximum for equal radiative and intrinsic damping rates. Overall the maximum field enhancement for the lossless case is around 7.5 times larger than for the lossy case. **i**, Comparison between coupling strength and mean field enhancement in resonator structure with respect to the BIC Q factor without losses. The field enhancement scales with $Q^{1/2}$ and would saturate due to the square root at larger Q factors than shown. The coupling strength is saturating earlier with a differing functional dependence. **j**, The Rabi splitting and coupling strength plotted against the total quality factor for a lossy material. The functional dependence differs due to a change in quality factors, however the Rabi splitting and coupling strength still show the same trend.

**Supplementary Note 4: Polaritonic critical coupling**

The absorbance of the polariton branches in the strong coupling regime can be maximized when the intrinsic and radiative damping rates in the system are matched. Specifically, in the case of our TMDC BIC, one has to take the background absorption of the material into account (like in regular critical coupling scenarios[4]) as well as the additional intrinsic damping rate from the exciton. We studied the toy model from Supplementary Note 3 for different imaginary parts of the refractive index $\varepsilon_2$ from 0 to 0.5 and different exciton linewidths $\gamma_{\text{ex}}$ from around 1 to 11 meV. For different asymmetry parameters $\Delta L_{0,}$ the maximum absorbance value for our two-port system of 0.5 is reached for specific set of values of $\varepsilon_2$ and $\gamma_{\text{ex}}$ (**Fig. S10a**). The maximum absorption occurs whenever the radiative damping rate of the BIC is matched with its intrinsic damping rate influenced by $\varepsilon_2$ and the intrinsic damping rate of the exciton, i.e.

when the condition

$$\left|\gamma_{\text{rad,BIC}} - (\gamma_{\text{int,BIC}} + \gamma_{\text{int,Ex}})\right| = 0$$

is met (**Fig. S10b**). The BIC concept with its control over the radiative linewidth thus enables the easy acquisition of the optimal asymmetry parameter that propels the strongly coupled system into the critical coupling regime for a given set of values of background absorption and excitonic width (**Fig. S10c**)

To demonstrate this for a realistic material, we slightly altered the structure parameters of the BIC design from the main text to h = 70 nm and P0 = 320 nm. The simulations were carried out in CST with the TL material from the manuscript, which showed excellent agreement with our experimental results. The polaritonic critical coupling regime can be easily reached with asymmetry parameters around $\Delta L_0$ = 100 nm (**Fig. S10d**).

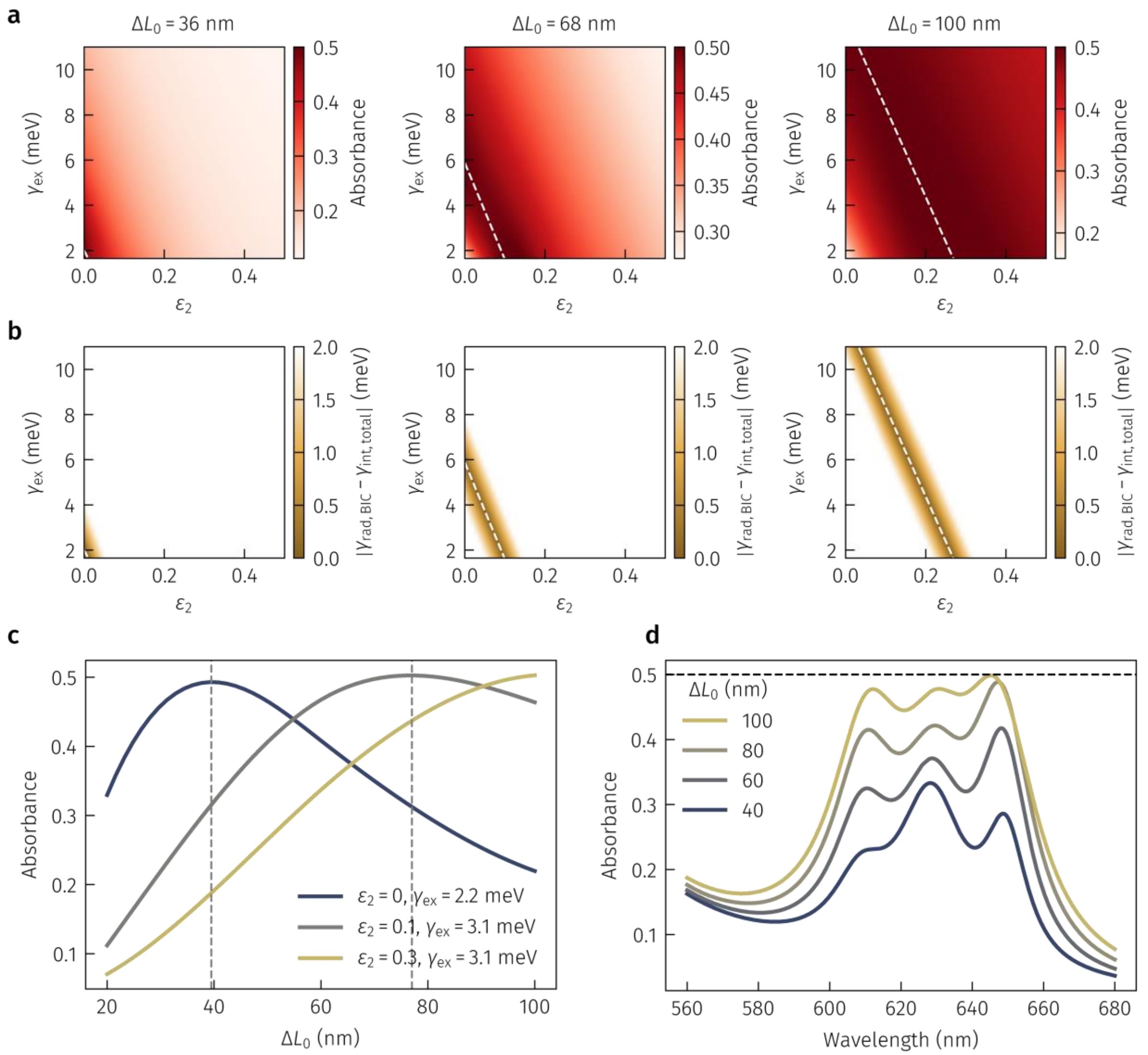


**Figure S10: Polaritonic critical coupling. a**, Extracted absorbance values averaged over both polariton branches for spectrally matched BIC and exciton in the strong coupling regime for different asymmetry parameters $\Delta L_0$ and **b**, the absolute value of the difference between the radiative damping rate of the BIC and the total intrinsic damping rate of the system. The white dashed line shows the condition of polaritonic critical coupling. **c**, Absorbance values with respect to the asymmetry parameter $\Delta L_0$ for different sets of background absorption and exciton width. **d**, The simulated absorbance spectra for our TL material from the main text with slightly altered structural parameters (h = 70 nm, P0 = 320 nm) reach the polaritonic critical coupling regime for $\Delta L_0$ ~ 100 nm.

## Supplementary Note 5: Estimation of mode volume and confinement factor

To calculate the modal volume of a BIC resonance we used a generalized expression to account for non-negligible losses in the system[5]

$$\frac{1}{V_{eff}} = \mathrm{Re}\left\{\frac{1}{v}\right\}, v = \frac{\langle\langle \mathbf{E}(\mathbf{r}) | \mathbf{E}(\mathbf{r}) \rangle\rangle}{\max\left(\varepsilon(\mathbf{r}) |\mathbf{E}(\mathbf{r})|^2\right)} \quad \text{(S9)}$$

The inner product consists of a volume integral term and a flux integral to account for the varying degree of radiative loss for the BIC platform[6]

$$\langle\langle \mathbf{E}(\mathbf{r}) | \mathbf{E}(\mathbf{r}) \rangle\rangle = \int_V \varepsilon(\mathbf{r}) |\mathbf{E}(\mathbf{r})|^2 \, d\mathbf{r} + \frac{c^2}{2\omega} \oint_{\partial V} \Phi(\mathbf{r}) \cdot d\mathbf{S} \quad \text{(S10)}$$

with

$$\Phi(\mathbf{r}) = -\frac{1}{2}\nabla E^2 - k^2 \mathbf{r} E^2 + \mathbf{r} \sum_{ij} \left(\frac{\partial E_i}{\partial x_j}\right)^2 - 2\sum_i K_i \nabla E_i$$

$$\mathbf{K}(\mathbf{r}) = (\mathbf{r} \cdot \nabla)\mathbf{E}(\mathbf{r}) \quad \text{(S11)}$$

where $k$ is the wavevector in free space and $\omega$ the complex frequency of the cavity mode.

We simulated the electric near fields in one unit cell with Lumerical, assuming a homogeneous environment and a constant permittivity of the resonator material of $\varepsilon = 10$, using a fixed mesh and periodic boundary conditions. To assure an accurate result, we increased the z-dimension of the simulation domain until the mode volume converged against a constant value for each value of the asymmetry parameter $\Delta L_0$.

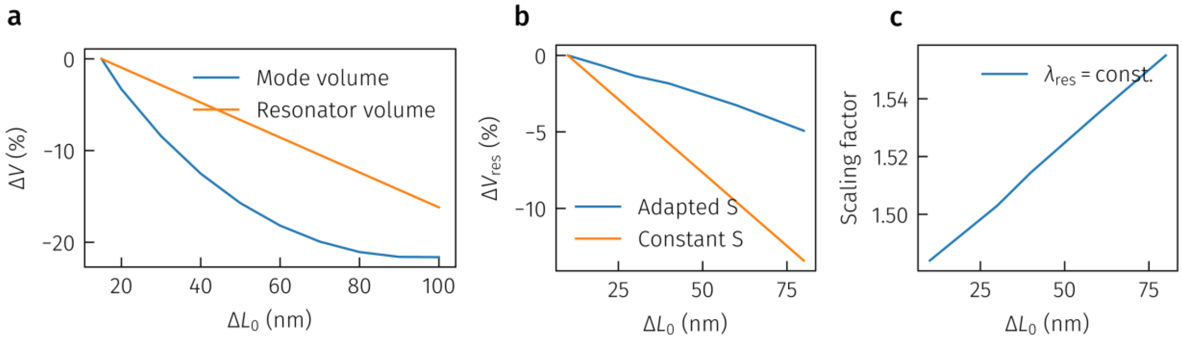


**Figure S11: Effective mode volumes for TMDC BICs. a**, Relative change in effective mode volume

calculated according to and resonator volume for different asymmetry factors for constant scaling factor. **b**, Relative change in the resonator volume for constant scaling factor and scaled unit cell to maintain the same BIC resonance wavelength. **c**, Unit cell scaling factor for different asymmetry parameters to maintain a constant resonance wavelength.

Likewise, we calculated the optical confinement factor Γ for our BIC structure based on the dielectric displacement calculated via $\mathbf{D}(\mathbf{r}) = \varepsilon(\mathbf{r})\mathbf{E}(\mathbf{r})$

$$\Gamma = \frac{\int_{V_{\text{structure}}} |\mathbf{D}(\mathbf{r})|^2 \, \mathrm{d}\mathbf{r}}{\int_{V_{\mathbf{total}}} |\mathbf{D}(\mathbf{r})|^2 \, \mathrm{d}\mathbf{r}}$$

for a non-dispersive, anisotropic dielectric defined as

$$\varepsilon = \begin{pmatrix} 10 + \varepsilon_2 i & 0 & 0 \\ 0 & 10 + \varepsilon_2 i & 0 \\ 0 & 0 & 7 \end{pmatrix}$$

with varying imaginary parts of the permittivity using the structural parameters from the manuscript. Similar to our mode volume analysis, we tested the necessary integration volume for convergence.

As can be seen from Fig. S12, for lossless materials ($\varepsilon_2$ = 0) the value of the confinement factor Γ starts above 90% and decreases slightly with increasing asymmetry factor $\Delta L_0$. Including losses, for small asymmetries the confinement factor is significantly reduced, rises rapidly with increasing asymmetry parameter and converges towards a mutual decreasing trend. The value $\varepsilon_2$ = 0.6 corresponds to the losses present at 629 nm without exciton (Fig. 1b), where the confinement factor peaks at $\Delta L_0 = 100$ nm with approx. 83%.

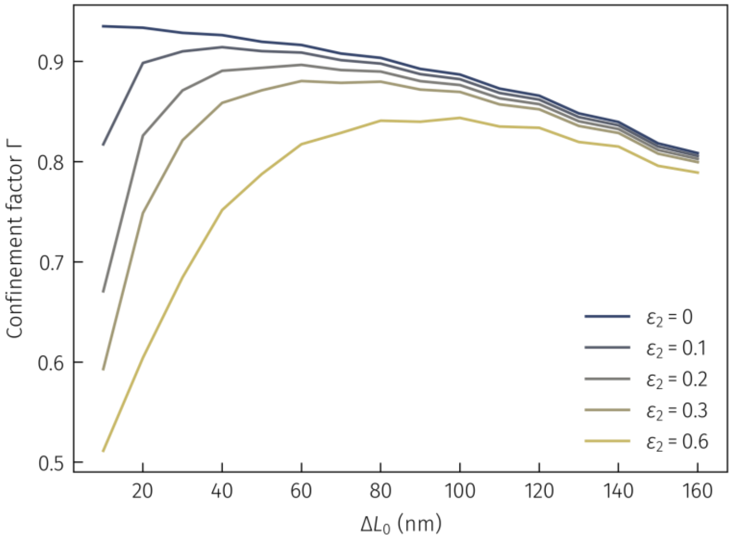


**Figure S12: Optical confinement factor for different imaginary parts of the permittivity.** The value $\varepsilon_2 = 0.6$ corresponds to the losses present at 629 nm without exciton (see Fig. 1b in the main text).

**Supplementary Note 5: Comparison with other strong-coupling implementations**

| **Reference** | **Type of structure** | **TMDC structure** | **Coupling type** | **Straightforward control over Q factor** | **Rabi splitting** |
|---|---|---|---|---|---|
| [7] | Dielectric | Bulk | Intrinsic | No | 190 meV |
| [8] | Hybrid | Bulk | Intrinsic+Evanescent | No | 410 meV |
| [9] | Plasmonic | Monolayer - Multilayer | Evanescent | No | 140 meV |
| [10] | Dielectric | Monolayer | Evanescent | Yes | 27 meV |
| Our work | Dielectric | Bulk | Intrinsic | Yes | 116 meV |

**Table S2: Comparison with other strong-coupling implementations**

**References**

1. Jung, G.-H., Yoo, S. & Park, Q.-H. Measuring the optical permittivity of two-dimensional materials without a priori knowledge of electronic transitions. *Nanophotonics-berlin* 8, 263–270 (2018).

2. Haus, H. A. *Waves and Fields in Optoelectronics*. (Prentice-Hall, 1984).

3. Cao, S. *et al.* Normal-Incidence-Excited Strong Coupling between Excitons and Symmetry-Protected Quasi-Bound States in the Continuum in Silicon Nitride–WS2 Heterostructures at Room Temperature. *J Phys Chem Lett* 11, 4631–4638 (2020).

4. Adato, R., Artar, A., Erramilli, S. & Altug, H. Engineered Absorption Enhancement and Induced Transparency in Coupled Molecular and Plasmonic Resonator Systems. *Nano Lett* 13, 2584–2591 (2013).

5. Kristensen, P. T., Vlack, C. V. & Hughes, S. Generalized effective mode volume for leaky optical cavities. *Opt Lett* 37, 1649 (2012).

6. Muljarov, E. A. & Langbein, W. Exact mode volume and Purcell factor of open optical systems. *Phys Rev B* 94, 235438 (2016).

7. Verre, R. *et al.* Transition metal dichalcogenide nanodisks as high-index dielectric Mie nanoresonators. *Nat Nanotechnol* 14, 679–683 (2019).

8. Zhang, H. *et al.* Hybrid exciton-plasmon-polaritons in van der Waals semiconductor gratings. *Nat Commun* 11, 3552 (2020).

9. Kleemann, M.-E. *et al.* Strong-coupling of WSe2 in ultra-compact plasmonic nanocavities at room temperature. *Nat Commun* 8, 1296 (2017).

10. Kravtsov, V. *et al.* Nonlinear polaritons in a monolayer semiconductor coupled to optical bound states in the continuum. *Light Sci Appl* 9, 56 (2020).
